# Cuproptosis-related lncRNAs potentially predict prognosis and therapy sensitivity of breast cancer

**DOI:** 10.3389/fphar.2023.1199883

**Published:** 2023-07-17

**Authors:** Xiwen Wu, Ying Zhang, Gehao Liang, Huizhen Ye

**Affiliations:** ^1^ Department of Clinical Nutrition, Sun Yat-sen University Cancer Center, State Key Laboratory of Oncology in South China, Collaborative Innovation Center for Cancer Medicine, Guangzhou, China; ^2^ Staff and Faculty Clinic, Sun Yat-sen University Cancer Center, State Key Laboratory of Oncology in South China, Collaborative Innovation Center for Cancer Medicine, Guangzhou, China; ^3^ Department of Breast Surgery, Sun Yat-sen University Cancer Center, State Key Laboratory of Oncology in South China, Collaborative Innovation Center for Cancer Medicine, Guangzhou, China

**Keywords:** cuproptosis, lncRNA, biological functions, clinical practice, breast cancer

## Abstract

**Background:** Cuproptosis-related lncRNAs regulate the biological functions of various cancers. However, the role of cuproptosis-related lncRNAs in breast cancer remains unclear. In this study, we investigated the biological functions and clinical applications of cuproptosis-related lncRNAs in breast cancer.

**Methods:** The Cancer Genome Atlas (TCGA) database and the GSE20685 dataset were used for screening cuproptosis-related lncRNAs. Colony formation and CCK-8 kit assays were performed for detecting the proliferative function of cuproptosis-related lncRNAs, whereas wound healing, migration, and invasion assays were performed for detecting the metastatic regulation of cuproptosis-related lncRNAs in breast cancer. Finally, a prognostic cuproptosis-related lncRNA model was constructed using LASSO Cox regression analysis for detecting survival and sensitivity to conventional treatment (endocrine therapy, chemotherapy, and radiotherapy) and novel therapy (PARP and CDK4/6 inhibitors).

**Results:** In this study, we screened six cuproptosis-related lncRNAs associated with the survival of patients with breast cancer. Biofunctional experiments indicated that cuproptosis-related lncRNAs play essential roles in regulating the proliferation and metastasis of breast cancer cells. Finally, we applied a model of six cuproptosis-related lncRNAs to classify the patients into high- and low-risk groups. High-risk group patients exhibited worse survival rates (*p* < 0.001) and lower sensitivity to chemotherapy, endocrine therapy, and radiation therapy. Compared with high-risk patients, low-risk patients exhibited a lower expression of CDK4/6 inhibitor-resistant biomarkers (CCNE1, E2F1, and E2F2) and PARP inhibitor-resistant biomarkers (*BRCA1*/*BRCA2*), indicating that patients in the low-risk group were more suitable for PARP inhibitor and CDK4/6 inhibitor application.

**Conclusion:** Cuproptosis-related lncRNAs are essential for regulating the biological functions of breast cancer, and they have the potential to predict prognosis and sensitivity of breast cancer to various therapies.

## 1 Introduction

A recent global cancer statistic reported that female breast cancer (BC) has now surpassed lung cancer as the leading cause of global cancer incidence in 2020 and become the fifth leading cause of cancer mortality worldwide (Yersal and Barutca, 2014). As a heterogeneous cancer, the prognosis of BC is affected by the pathological stage, immunohistochemical subtypes, and multiagent treatments (Weigel and Dowsett, 2010). However, patients with BC presenting the same TNM stage and immunohistochemical subtype exhibit various prognoses after standard treatments owing to different sensitivities to therapy (Yersal and Barutca, 2014). Numerous studies have indicated that cancer susceptibility genes (CSGs) play important roles in regulating biological functions and therapeutic resistance of cancers (Weigel and Dowsett, 2010; Shi et al., 2022a).

Copper is an essential trace element that ensures the normal growth of the body and physiological functions. However, excess copper is toxic and leads to cell death by binding to the lipoylated components of the tricarboxylic acid (TCA) cycle. The copper compound then induces lipoylated protein aggregation, subsequent iron–sulfur cluster protein loss, proteotoxic stress, and ultimately cell death, in a process called cuproptosis (Tsvetkov et al., 2022). Copper metabolism was reported to regulate tumor progression, including tumor microenvironment formation, angiogenesis, metastasis, and proliferative immortality (Blockhuys and Wittung-Stafshede, 2017; Blockhuys et al., 2020). In addition, copper metabolism plays an important role in regulating anti-cancer activities. For example, copper supplements enhance the expression of PD-L1, which induced cancer immune escape. Copper chelating agents increased tumor-infiltrating CD8 T cells and natural killer cells and slowed tumor growth (Voli et al., 2020). Higher serum copper levels have been found in patients with BC than in healthy individuals or patients with benign breast diseases (Feng et al., 2020). In addition, copper has a high affinity for estrogen receptors, and it induces estrogen-regulated pathways (Kulkoyluoglu-Cotul et al., 2019). In the last decade, two main therapeutic strategies based on copper-induced cell death were carried out. One is to use the corresponding copper chelating agent to reduce the bioavailability of copper so as to inhibit progression of tumor cells. The other is to provide excessive copper to induce cuproptosis of cancer cells. In this strategy, tumor-targeted nanoparticles based on copper delivery may be a very promising research direction, which not only retains the therapeutic effect of copper-induced cell death but also avoids the high systemic toxicity (Pramanik et al., 2017).

Long noncoding RNAs (lncRNAs) are an essential class of RNA molecules with lengths greater than 200 base pairs. LncRNAs play crucial roles in the biological functions of BC cells, including tumorigenesis, metabolism, proliferation, migration, invasion, and drug sensitivity, indicating their potential as diagnostic, prognostic, and therapeutic biomarkers (Schmitt and Chang, 2016; Wanowska et al., 2022; Ye et al., 2022). Cuproptosis-related lncRNA AC099850.3 is a prognostic marker for hepatocellular carcinoma (HCC) and has been confirmed to promote HCC progression via the PRR11/PI3K/AKT axis (Wang et al., 2021; Pak et al., 2022; Zhong et al., 2022). Upregulation of cuproptosis-related lncRNA NIFK-AS1 increases resistance to chemotherapy drugs through m6A methylation and promotes progression of HCC (Chen et al., 2021a; Pak et al., 2022). Furthermore, NIFK-AS1 suppresses M2-like polarization of macrophages in endometrial cancer. Thus, cuproptosis-related lncRNAs may play an essential role in carcinoma progression and regulate immune response or drug sensitivity of carcinoma.

A few cuproptosis-related models have been constructed and exhibited potential in predicting the survival and immune microenvironment of patients with BC (Jiang et al., 2022; Zhang et al., 2022); however, these models did not consider clinical characteristics and sectionalization. Moreover, previous models only performed validation in the same cohort used for model construction. Currently, several emerging drugs, such as CDK4/6 inhibitors (Wu et al., 2020) and poly (ADP-ribose) polymerase (PARP) inhibitors (Rose et al., 2020), have gradually become the first-line treatment for BC; however, few models have been developed for predicting the efficacy of these drugs. Therefore, it is important to develop a multifunctional model for predicting survival and therapeutic efficacy.

In this study, we identified six cuproptosis-related lncRNAs that play important roles in the regulation of BC cell proliferation and metastasis. We constructed a prognostic model based on these lncRNAs and used it to classify the patients into high- and low-risk groups. Patients in the high-risk group exhibited worse survival rates and poorer sensitivity to chemotherapy, endocrine therapy, and radiation therapy than those in the low-risk group. The gene expression of *BRCA1*/*BRCA2* was low in the low-risk group, while those of *TRIPI3*/*PARP1* and *MUS81* were high and low, respectively, in the high-risk group, indicating that patients in the low-risk group were more suitable for PARP inhibitor application and that patients in the high-risk group had a higher risk of drug resistance.

## 2 Materials and methods

### 2.1 Dataset preparation

Clinical information and corresponding RNA-sequencing data of 1,109 breast cancer patients were obtained from the TCGA database at the website of https://portal.gdc.cancer.gov/. After patients were excluded for no stage information, 1,054 patients from TCGA were used as the training cohort. A total of 317 patients selected randomly from TCGA with the R package “caret” were used as the internal validation cohort. The GSE20685 dataset (series matrix files) including 327 breast cancer patients from the platform GPL570 [HG-U133_Plus_2] Affymetrix Human Genome U133 Plus 2.0 Array (https://www.ncbi.nlm.nih.gov/geo/query/acc.cgi?acc=GSE20685) was used as the external validation cohort.

### 2.2 Identification of cuproptosis-related lncRNAs in breast cancer

A total of 1,109 tumor samples and 113 normal samples of RNA-sequencing data were screened with a false discovery rate (FDR) < 0.05 and |log2FC| ≥ 1. We used the ensemble human genome browser GRH38.p13 (http://asia.ensembl.org/index.html) to classify lncRNAs and protein-coding genes. Genetic information of 42 genes were gathered from previous literatures (Chen et al., 2022; Tsvetkov et al., 2022). The R package “limma” was used to calculate cuproptosis-related lncRNAs. With correlation coefficient |R2| > 0.3 and *p* < 0.001, 538 cuproptosis-related lncRNAs were obtained. Then, a total of 90 shared cuproptosis-related lncRNAs were extracted from TCGA and GSE20685 for the following analysis. All RNA-sequencing data were normalized by log2 conversion.

### 2.3 Cuproptosis-related lncRNA signature and model construction

Univariate Cox regression analysis and LASSO Cox regression analysis were used to calculate predictive cuproptosis-related lncRNAs and establish the cuproptosis-related lncRNA signature. The risk score of every cuproptosis-related lncRNA signature was calculated according to the following formula: Risk score = (Expi × βi), where “β” is the regression coefficient of every corresponding lncRNA and “exp” is the expression level of lncRNA.

Combining the lncRNA signature score with the clinical characteristics of age, gender, and stage, we used multivariate Cox regression analysis to construct a comprehensive model with the TCGA dataset and developed a nomogram to predict 1-, 3-, and 5-year survival probability. The R package “nomogramEx” was used to calculate the corresponding score of every factor in the comprehensive model. Then, the patients were classified into the high-risk group and low-risk group according to the median comprehensive model risk score.

### 2.4 Cell lines and siRNA transfection

The BC cell line MCF7 was cultured in RPMI-1640 (Gibco, United States) containing 10% fetal bovine serum (FBS) (Gibco, United States) and 1% penicillin/streptomycin (Gibco, United States). All cell lines were incubated at 37 °C and with 5% CO_2_. The small interfering RNA (siRNA) was designed for knocking down lncRNAs using Lipofectamine^®^ 3000 according to reagent instructions.

### 2.5 Cell proliferation assay

Cells were seeded in 96-well plates at a concentration of 3,000 cells per well in 200 μL of the medium. After cultivation for the indicated time, the medium was removed and 20 μL diluted CCK-8 solutions (Dojindo, Japan) were added. The plate was continued to be incubated at 37 °C for 2 h, and the absorbance was measured at 450 nm. All experiments were performed in three independent cohorts.

### 2.6 Colony formation assays

Cells were seeded in six-well plates at a concentration of 500 cells per well. The cell culture medium was changed every 3 days. After 2 weeks of culture, the medium was removed and the colonies were fixed in 10% formalin solution for 2 h, with 0.1% crystal violet solution staining subsequently for another 10 min at room temperature. Digital images of the plates were taken for permanent records, with colony counting done by ImageJ software (version 1.8.0).

### 2.7 Cell migration and invasion assays

Briefly, 5 × 10^4^ cells in 200 μL of RPMI-1640 (with 0% FBS) were seeded in the upper chambers of transwell membranes (Corning, New York, United States). The bottom wells of the chambers were filled with 800 μL RPMI-1640 (with 10% FBS). After 24 h of incubation, the transwell membranes were fixed with 95% ethanol and then stained with 1% crystal violet. Images of five different fields were captured from each membrane, and the number of migrated cells was counted. For cell invasion assays, the upper wells were pre-coated with Matrigel, and other procedures of invasion assays were similar with those of migration assays.

### 2.8 Gene set enrichment analysis

Gene set enrichment analysis (GSEA; http://www.broadinstitute.org/gsea) version 4.1.0 (Broad Institute, United States) was used to analyze the genes that were differently expressed between the high- and low-risk groups. False discovery rate (FDR q-value) < 0.25 and |NSE| < 2 and normal *p*-value < 0.05 were considered significantly enriched. The R packages “plyr,” “ggplot2,” “grid,” and “gridExtra” were used to conduct the multi-GSEA analysis.

### 2.9 Estimation of tumor-infiltrating immune cells

The CIBERSORT algorithm was used to calculate the proportion of 22 different tumor-infiltrating immune cells in high- and low-risk groups. The normalized expression data were available at the CIBERSORT website (http://cibersort.stanford.edu/). Bar plot, heatmap, corHeatmap, and vioplot were drawn by R package, showing the association between different immune cells and discrepancy between the two groups.

### 2.10 Statistical analysis

All statistical analysis and figures were performed by R software, GraphPad Prism 5 Software, and SPSS 22.0. Kaplan–Meier plotter was used to evaluate the discrepancy of overall survival (OS) between the high-risk group and the low-risk group in different subgroup patients. The chi-squared test was used to compare the proportions of clinical characteristics between the training cohort and two different validation cohorts. The cox proportional risk model was used to estimate hazard ratios (HRs) and 95% confidence intervals (CIs) of different age, TNM stage, tumor size (T), lymph node metastasis (N), distant metastasis (M), HER2 status, HR status, and carcinoma type between the two groups. Calibration curve was used to test the consistency between the predicted survival and actual survival of the comprehensive model. The time-dependent ROC curve analysis of different groups was performed with R packages “survivalROC” and “timeROC.” The Wilcoxon test was used to compare the proportion of immune cells and the biomarker expression levels of emerging therapy between the two groups. *p*-value < 0.05 was considered statistically significant. All *p* values were two-tailed.

## 3 Results

### 3.1 Screening cuproptosis-related lncRNAs in The Cancer Genome Atlas (TCGA) database

In total, 1,054 patients with BC after non-stage exclusion in the TCGA database were enrolled as the training cohort for screening prognostic lncRNA candidates and identifying cuproptosis-related lncRNA signatures. A total of 317 patients randomly selected from the training cohort were included in the internal validation cohort. In addition, 327 patients with BC from GSE20685 were enrolled as an external validation cohort. The detailed clinical characteristics of the three cohorts are presented in Table 1. No significant differences in clinical characteristics were found between the training and internal validation cohorts, whereas a significant difference was found between the training and external validation cohorts.

**TABLE 1:** Clinical characteristics of patients in training and validation cohorts.

Variable	Training cohort (N = 1054) (%)	Internal validation cohort (N = 317) (%)	*p*	External validation cohort (n = 327)	*p*
Age			0.31		<0.001
≤40 years	75 (7.12%)	28 (8.83%)		71 (21.72%)	
>40 years	979 (92.88%)	289 (91.17%)		256 (78.29%)	
Gender			0.77		0.05
Female	1,042 (98.86%)	314 (99.05%)		327 (100%)	
Male	12 (1.14%)	3 (0.95%)		0 (0%)	
Stage			0.96		<0.001
1	181 (17.17%)	55 (17.35%)		69 (21.10%)	
2	609 (57.78%)	185 (58.36%)		147 (44.95%)	
3–4	264 (25.05%)	77 (24.29%)		111 (33.95%)	
T			0.99		0.07
T1	273 (25.90%)	82 (25.87%)		101 (30.89%)	
T2	615 (58.35%)	184 (58.04%)		188 (57.49%)	
T3	132 (12.52%)	40 (12.62%)		26 (7.95%)	
T4	34 (3.23%)	11 (3.47%)		12 (3.67%)	
M			0.854		<0.001
M0	885 (83.97%)	270 (85.17%)		319 (97.55%)	
M1	23 (2.18%)	7 (2.21%)		8 (2.45%)	
NA	146 (13.85%)	40 (12.62%)		0 (0%)	
N			0.94		<0.001
N0	500 (47.44%)	153 (48.26%)		137 (41.90%)	
N1	355 (33.68%)	109 (34.38%)		87 (26.60%)	
N2	119 (11.29%)	35 (11.04%)		63 (19.27%)	
N3	71 (6.74%)	17 (5.36%)		40 (12.23%)	
NA	9 (0.85%)	3 (0.94%)		0 (0%)	
ER			0.40		
Positive	775 (73.53%)	241 (76.03%)			
Negative	232 (22.01%)	67 (21.14%)			
NA	47 (4.46%)	9 (2.83%)			
PR			0.45		
Positive	673 (63.85%)	209 (65.93%)			
Negative	331 (31.40%)	98 (30.91%)			
NA	50 (4.74%)	10 (3.15%)			
HER2			0.30		
Negative	738 (70.02%)	227 (71.61%)			
Positive	187 (17.74%)	61 (19.24%)			
NA	129 (12.24%)	29 (9.15%)			
Therapy					
Endocrinotherapy	513 (48.67%)	150 (47.32%)	0.67		
Chemotherapy	573 (54.36%)	180 (56.78%)	0.45		
Radiation therapy	518 (49.15%)	157 (49.53%)	0.91		

**FIGURE 1:**
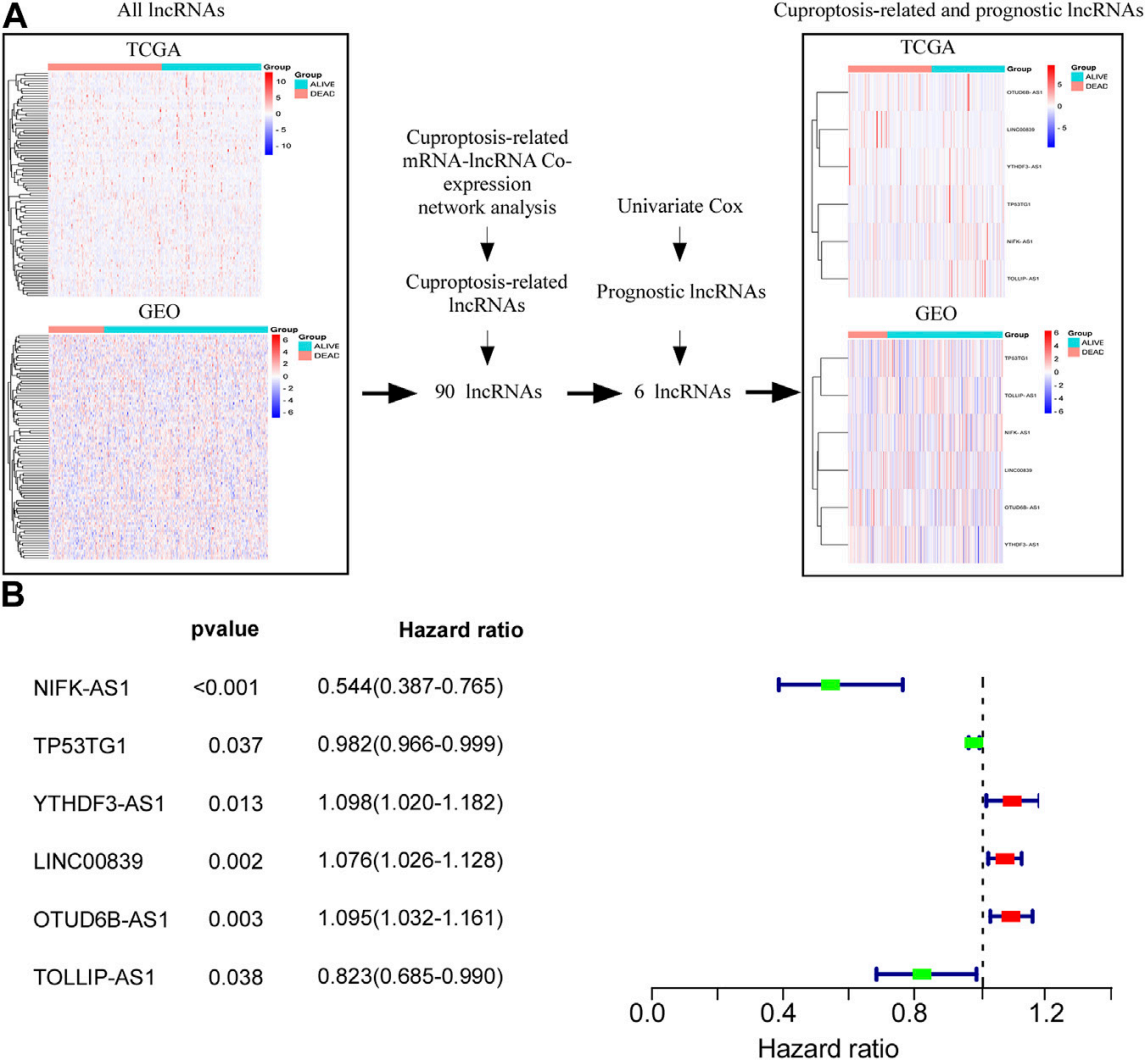
Screening cuproptosis-related lncRNAs in TCGA and GSE20685 databases. **(A)** Screening of six lncRNAs (NIFK-AS1, TP53TG1, TOLLIP-AS1 YTHDF3-AS1, LINC00839, and OTUD6B-AS1) which were associated with prognosis of BC patients. **(B)** Forest map of univariate Cox regression analysis.

Supplementary Figure S1 presents a flowchart of the cuproptosis-related comprehensive model construction. LncRNAs with an absolute Pearson correlation coefficient of 0.3 (|R| > 0.3) and *p* < 0.001 were considered cuproptosis-related lncRNAs. A total of 90 shared lncRNAs were extracted from the cuproptosis-related lncRNAs from TCGA and GSE20685 for subsequent analyses (Figure 1A). Univariate Cox regression analysis was used for selecting six lncRNAs (NIFK-AS1, TP53TG1, TOLLIP-AS1, YTHDF3-AS1, LINC00839, and OTUD6B-AS1) that were associated with the prognosis of patients with BC (Figure 1A). Figure 1B depicts a forest map of the univariate Cox regression analysis.

### 3.2 Biological functions of cuproptosis-related lncRNAs in BC cells

**FIGURE 2:**
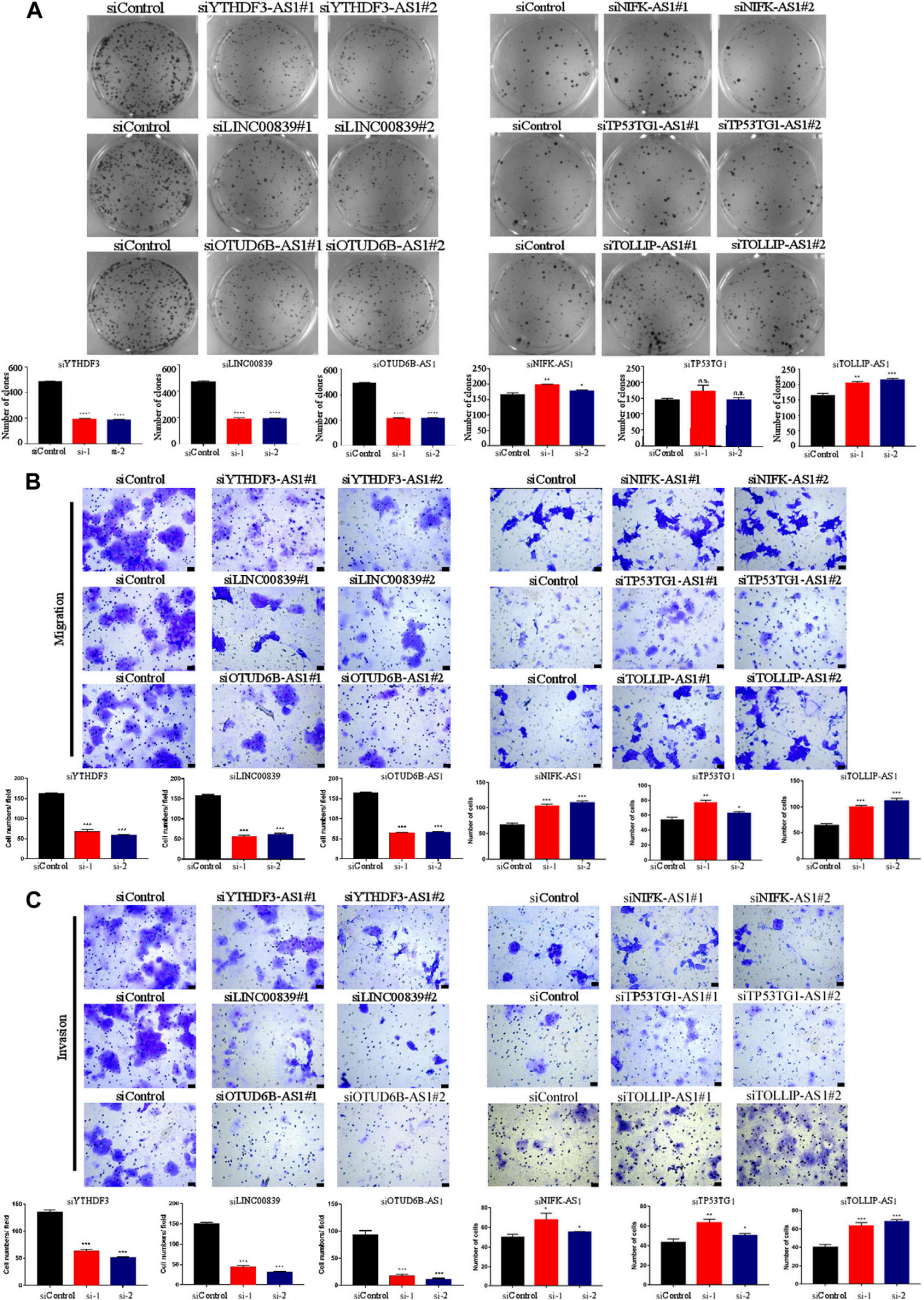
Biological functions of cuproptosis-related lncRNAs in BC cells. **(A)** Plate colony formation assay showed that colony formation of MCF-7 cells decreased after silencing YTHDF3-AS1, LINC00839, and OTUD6B-AS and increased after silencing NIFK-AS1 and TOLLIP-AS1. **(B)** The transwell migration experiment showed that knocking down YTHDF3-AS1, LINC00839, and OTUD6B-AS1 led to decrease of invasive MCF-7 cells, but increased after silencing NIFK-AS1, TP53TG1, and TOLLIP-AS1,—100 μm. **(C)** The transwell invasion experiment showed that knocking down YTHDF3-AS1, LINC00839, and OTUD6B-AS1 led to decrease of invasive MCF-7 cells, but increased after silencing NIFK-AS1, TP53TG1, and TOLLIP-AS1, scale bar—100 μm *p < 0.05, **p < 0.01, and ***p < 0.001, n.s. no significance, according to Student’s *t*-test.

We identified the biological functions of six cuproptosis-related lncRNAs in BC. Plate colony formation assay showed that colony formation of MCF-7 cells decreased after silencing YTHDF3-AS1, LINC00839, and OTUD6B-AS and increased after silencing NIFK-AS1 and TOLLIP-AS1 (Figure 2A). CCK-8 kit assay indicated that similar results as those of plate colony formation assay in MCF-7 cells were observed (Supplementary Figure S2A), indicating that the lncRNAs YTHDF3-AS1, LINC00839, and OTUD6B-AS1 played an important role in promoting BC cell proliferation. In addition, transwell migration and invasion experiment showed that knocking down YTHDF3-AS1, LINC00839, and OTUD6B-AS1 led to decrease of invasive MCF-7 cells, but increased after silencing NIFK-AS1, TP53TG1, and TOLLIP-AS1 (Figures 2B, C). A similar result was obtained by performing wound healing assay experiments (Supplementary Figure S2B). These results indicate that carcinogenesis-related lncRNAs exhibit significant potential in regulating BC metastasis.

### 3.3 Cuproptosis-related lncRNA signature construction

**FIGURE 3:**
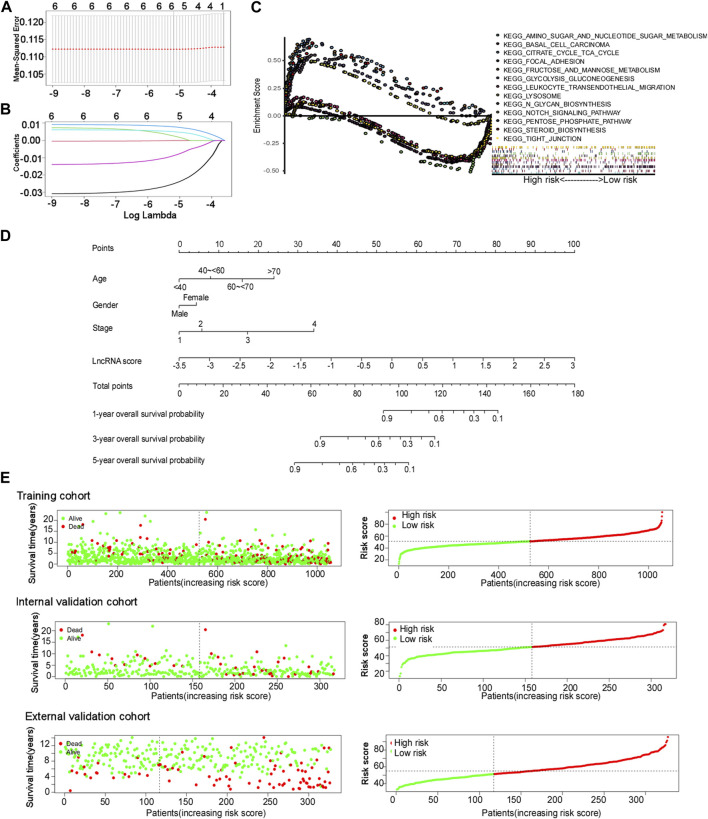
Cuproptosis-related lncRNA signature construction. **(A and B)** Least absolute shrinkage and selection operator (LASSO) regression performed with the minimum criteria. **(C)** Gene set enrichment analysis (GSEA) based on the cuproptosis-related comprehensive model. **(D)** Nomograms of 1-, 3-, and 5-year OS of BC patients by the cuproptosis-related comprehensive model. **(E)** Distribution of the risk score plot and the distribution of survival time in the training cohort, internal validation cohort, and external validation cohort.

Next, we determined the prognostic role of cuproptosis-related lncRNAs. In this study, three cohorts were enrolled (training cohort: 1,054 patients from the TCGA database, internal validation cohort: 317 patients from the TCGA database, external validation cohort: 327 patients from the GSE20685 dataset). Univariate and least absolute shrinkage and selection operator (LASSO) Cox regression analyses were performed for identifying lncRNA candidates and establish cuproptosis-related lncRNA signatures (Figures 3A, B). The cuproptosis-related lncRNA signature formula was as follows: Risk score = e (0.130 × YTHDF3.AS1 expression) + (0.054 × LINC00839 expression) + (0.056 × OTUD6B.AS1 expression) + (−0.487 × NIFK.AS1 expression) + (−0.006 × TP53TG1 expression) + (−0.145 × TOLLIP.AS1 expression).

Multivariate Cox regression analysis was used for constructing a comprehensive cuproptosis-related model and nomogram using age, sex, stage, and lncRNA score (Figure 3D). The age groups “<40, 40–60, 60–70, and >70” corresponded to “0, 7.98, 15.95, and 23.93” points, respectively. The scores for men and women were 0 and 4.41 points, respectively. The stages “1, 2, 3, and 4” corresponded to “0, 5.76, 17.24, and 34.09” points, respectively. LncRNA points were calculated using the following formula: lncRNA points = 15.385 × lncRNA score + 53.846. The total points of these four factors corresponded to different survival probabilities at 1, 3, and 5 years. The total points classified the patients with BC into two groups, with a median score of 51.27 points in the training cohort. The patients in the validation cohort shared the same model and calculation formula as those in the training cohort, and they were classified into two groups according to the same median score.

Gene set enrichment analysis (GSEA) was performed for exploring biological or signal transduction pathway dysfunctions by comparing the differences in lncRNA expression profiles between the high- and low-risk groups. The results showed that amino sugar and nucleotide sugar metabolism, fructose and mannose metabolism, citrate cycle, TCA cycle, glycolysis and gluconeogenesis, lysosomes, N-glycan biosynthesis, and steroid biosynthesis were enriched in the high-risk group, while the Notch signaling pathway, tight junction, focal adhesion, leukocyte transendothelial migration, and basal cell carcinoma were enriched in the low-risk group (Figure 3C).

### 3.4 Efficacy of the cuproptosis-related comprehensive model

The risk score plots, survival status plots, and heatmap of the six lncRNAs in high- or low-risk patients with BC in the three cohorts are displayed in Figure 3E and Supplementary Figure S3, indicating that as the risk increased, the number of deaths among patients with BC increased in all three cohorts. More patients died in the high-risk group than in the low-risk group.

**FIGURE 4:**
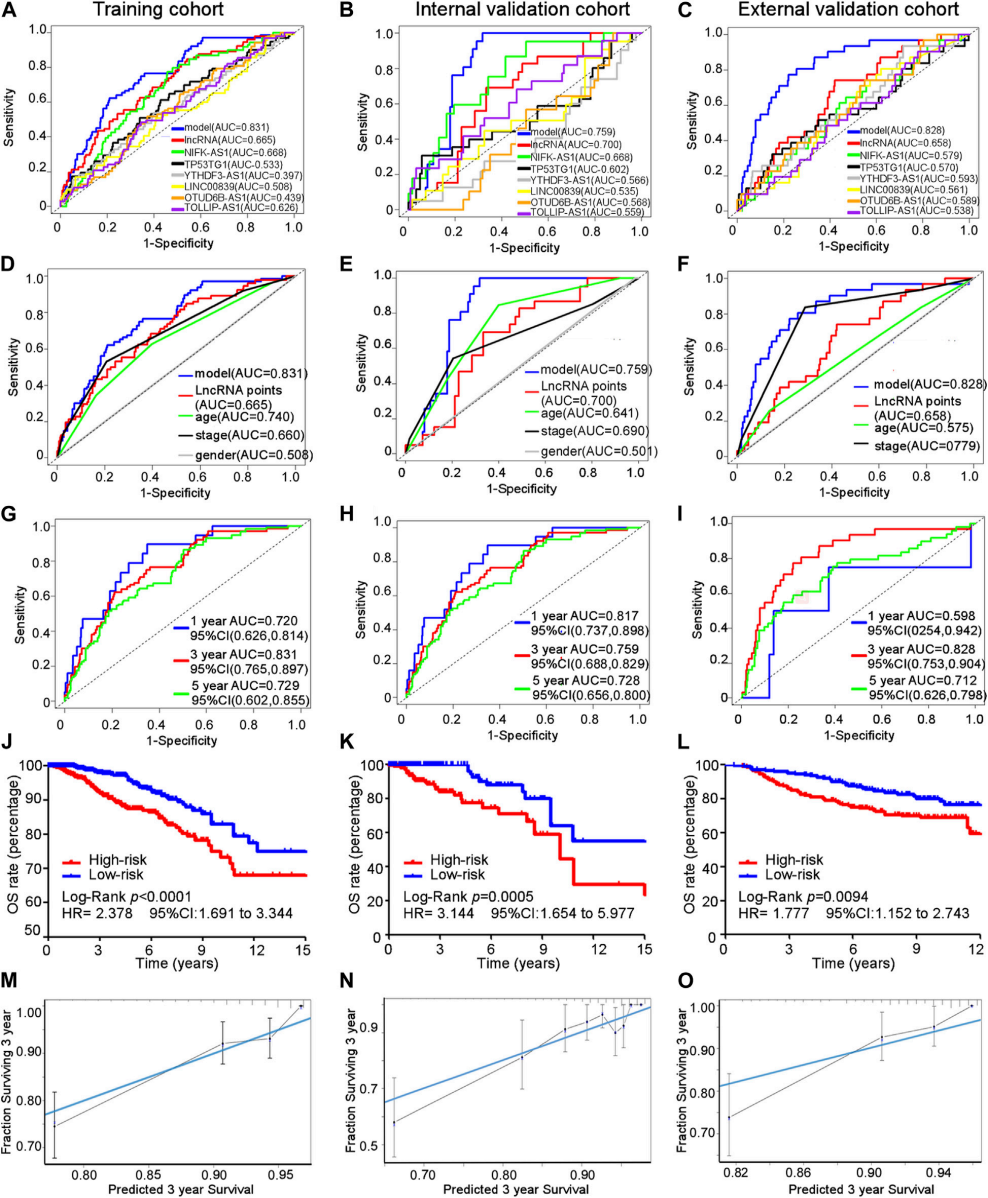
Efficacy of the cuproptosis-related comprehensive model. **(A–C)** AUC for different cuproptosis-related lncRNAs in training, internal, and external validation cohorts. **(D–F)** AUC of every predictive factor of the cuproptosis-related comprehensive model in training, internal, and external validation cohorts. **(G–I)** AUC of 1-, 3-, and 5-year survival probability of the cuproptosis-related comprehensive model in training, internal, and external validation cohorts. **(J–L)** Kaplan–Meier curves for the overall survival in training, internal, and external validation cohorts. **(M–O)** Calibration curves of the cuproptosis-related comprehensive model in training, internal, and external validation cohorts.

The area under the ROC curve (AUC) for the cuproptosis-related comprehensive model for predicting the 3-year overall survival (OS) was greater than that for single cuproptosis-related lncRNA or lncRNA signature in all three cohorts (Figures 4A–C). Regarding the ability to predict the 3-year OS, the AUC of the cuproptosis-related comprehensive model was superior to single clinical characteristics such as age, TNM stage, and sex in all three cohorts (Figures 4D–F). The OS values at 1, 3, and 5 years, predicted by the cuproptosis-related comprehensive model, were 0.720, 0.831, and 0.729, respectively, in the training cohort; 0.817, 0.759, and 0.728, respectively, in the internal validation cohort; and 0.598, 0.828, and 0.712, respectively, in the external validation cohort (Figures 4G–I). These results suggest the high efficacy of the cuproptosis-related comprehensive model in predicting the survival of patients with BC. Kaplan–Meier analysis of the OS in all three cohorts indicated that BC patients with higher risk scores exhibited worse OS performance than BC patients with lower risk scores (training cohort: median OS 9.51 years vs. 12.21 years, HR 2.378, 95% CI = 1.691–3.344, *p* < 0.0001). Internal validation cohort: median OS: 10.05 years vs. 18.06 years, HR 3.144, 95% CI = 1.654–5.977, *p* < 0.0005. External validation cohort: HR 1.777, 95% CI = 1.152–2.743, *p* = 0.0094) (Figures 4J–L). A similar result was found in another independent dataset GSE21653 (Supplementary Figure S5). The calibration curves of all three cohorts indicated good agreement between the actual OS rate and the OS rate predicted using our cuproptosis-related comprehensive model (Figures 4M–O).

### 3.5 Subgroup analysis of the cuproptosis-related comprehensive model in predicting OS in the training cohort

**FIGURE 5:**
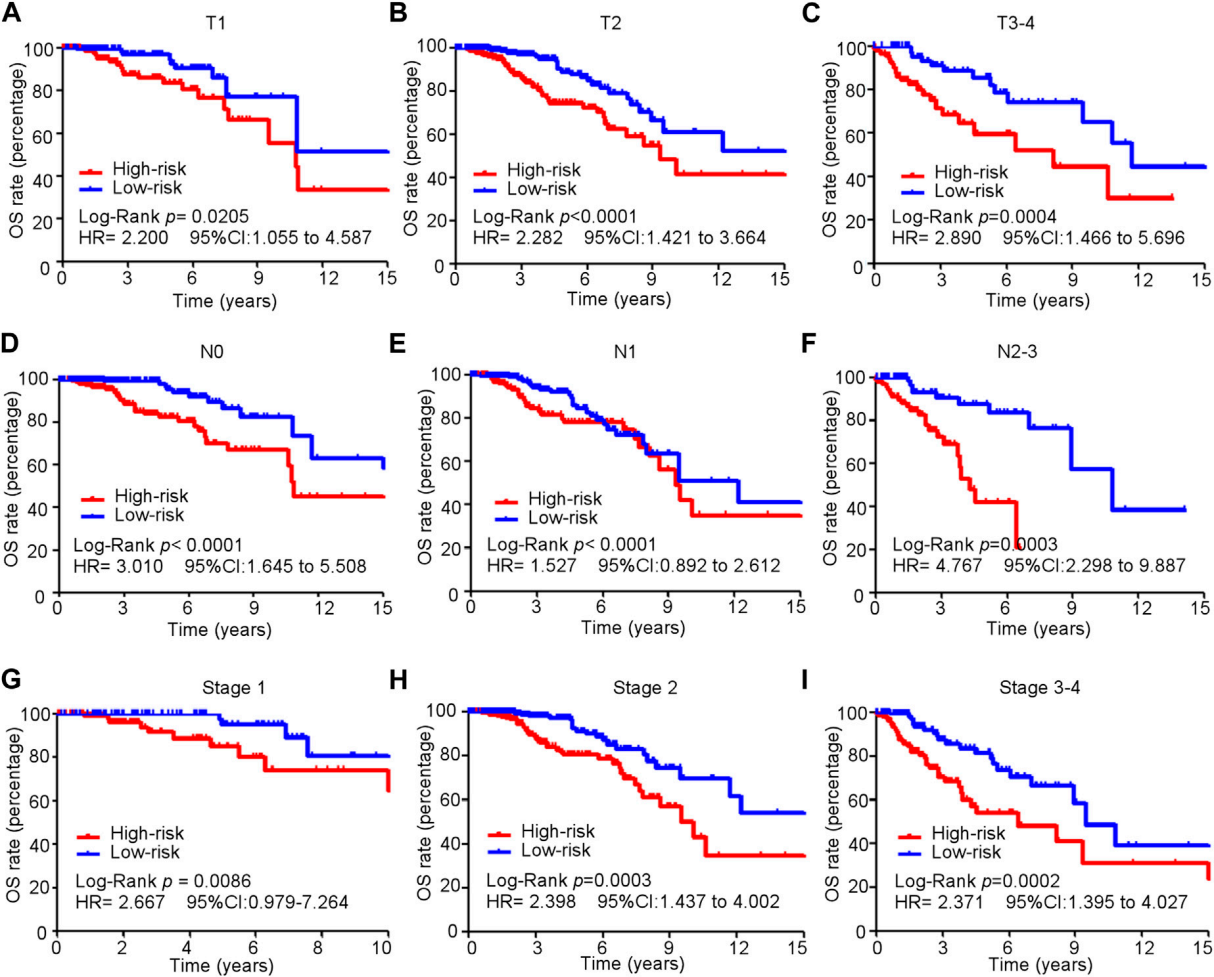
Subgroup analysis of the cuproptosis-related comprehensive model in predicting OS in the training cohort. **(A–I)** Kaplan–Meier curves for overall survival in all patients in different subgroups.

Kaplan–Meier and subgroup analyses of the cuproptosis-related comprehensive model were performed on the basis of the clinical characteristics of patients with BC in the training cohort (Table 2). Kaplan–Meier analysis showed that high-risk patients had poorer OS in the T1–T4, M0, and N0-3 subgroups than low-risk patients (Figures 5A–I). The *HER2* gene is an important risk factor and therapeutic target in patients with BC. Significantly more patients in the low-risk group survived longer than those in the high-risk group among patients, irrespective of HR+ HER2−, HR+ HER2+, HR− HER2+, or TNBC subtype (Supplementary Figure S4).

**TABLE 2:** Clinical characteristics of patients between high- and low-risk groups and in the training cohort.

Subgroup	Patients	High risk (%)	Low risk (%)	HR (95% CI)	*p*
All	1,054	527 (50.00%)	527 (50.00%)	2.378 (1.691–3.344)	<0.0001
Age					
≤40 years	96	6 (6.25%)	90 (93.75%)	2.038 (0.313–13.26)	0.456
>40 years	958	521 (54.38%)	437 (45.62%)	2.638 (1.838–3.786)	<0.0001
T					
T1	273	133 (48.72%)	140 (51.22%)	2.200 (1.055–4.587)	<0.0001
T2	615	314 (51.06%)	301 (48.94%)	2.282 (1.421–3.664)	<0.0001
T3-4	166	80 (48.19%)	86 (51.81%)	2.890 (1.466–5.698)	0.0004
NA	0				
N					
N0	500	232 (46.20%)	268 (53.80%)	3.010 (1.645–5.508)	<0.0001
N1	355	200 (56.34%)	155 (43.66%)	1.527 (0.892–2.612)	0.0124
N2-3	190	94 (50.00%)	96 (50.00%)	4.767 (2.298–9.887)	0.0003
NA	9				
M					
M0	885	452 (51.07%)	433 (48.93%)	2.399 (1.651–3.488)	<0.0001
M1	23	10 (43.48%)	13 (46.52%)	1.034 (0.384–2.787)	0.947
NA	137				
Stage					
1	181	91 (50.28%)	90 (47.72%)	2.667 (0.979–7.264)	0.0086
2	609	308 (49.59%)	301 (50.41%)	2.398 (1.437–4.002)	0.0003
3–4	264	136 (51.52%)	128 (48.48%)	2.371 (1.395–4.027)	0.0002
NA	16				
Molecular classification					
HR+, HER2+	147	61 (41.50%)	86 (58.50%)	4.989 (1.771–14.06)	0.0098
HR−, HER2+	38	12 (31.58%)	26 (68.52%)	2.431 (0.454–13.02)	0.2994
HR+, HER2−	690	366 (53.04%)	324 (46.96%)	2.090 (1.339–3.262)	<0.0001
Triple negative	157	78 (49.68%)	79 (50.32%)	2.969 (1.307–6.747)	0.0094
Type					
Infiltrating lobular carcinoma	196	106 (54.08%)	90 (45.92%)	3.994 (1.636–9.747)	0.0026
Infiltrating ductal carcinoma	755	366 (48.48%)	389 (51.52%)	2.258 (1.505–3.389)	<0.0001
Others	103	55 (53.40%)	48 (46.60%)	0.091 (0.860–5.674)	0.0825
Therapy					
Endocrine therapy	513	260 (50.68%)	253 (49.32%)	1.888 (0.973–3.666)	0.042
Chemotherapy	575	336 (58.43%)	239 (41.57%)	2.938 (1.551–5.566)	0.0002
Radiation therapy	518	268 (51.74%)	250 (48.26%)	2.559 (1.328–4.933)	0.0021

### 3.6 The immune cell infiltration landscape in breast cancer

The CIBERSORT algorithm was used in investigating the immune cell infiltration landscape in all three cohorts. A bar plot of the tumor-infiltrating cell proportions and heatmap of the tumor-infiltrating cells were generated.

**FIGURE 6:**
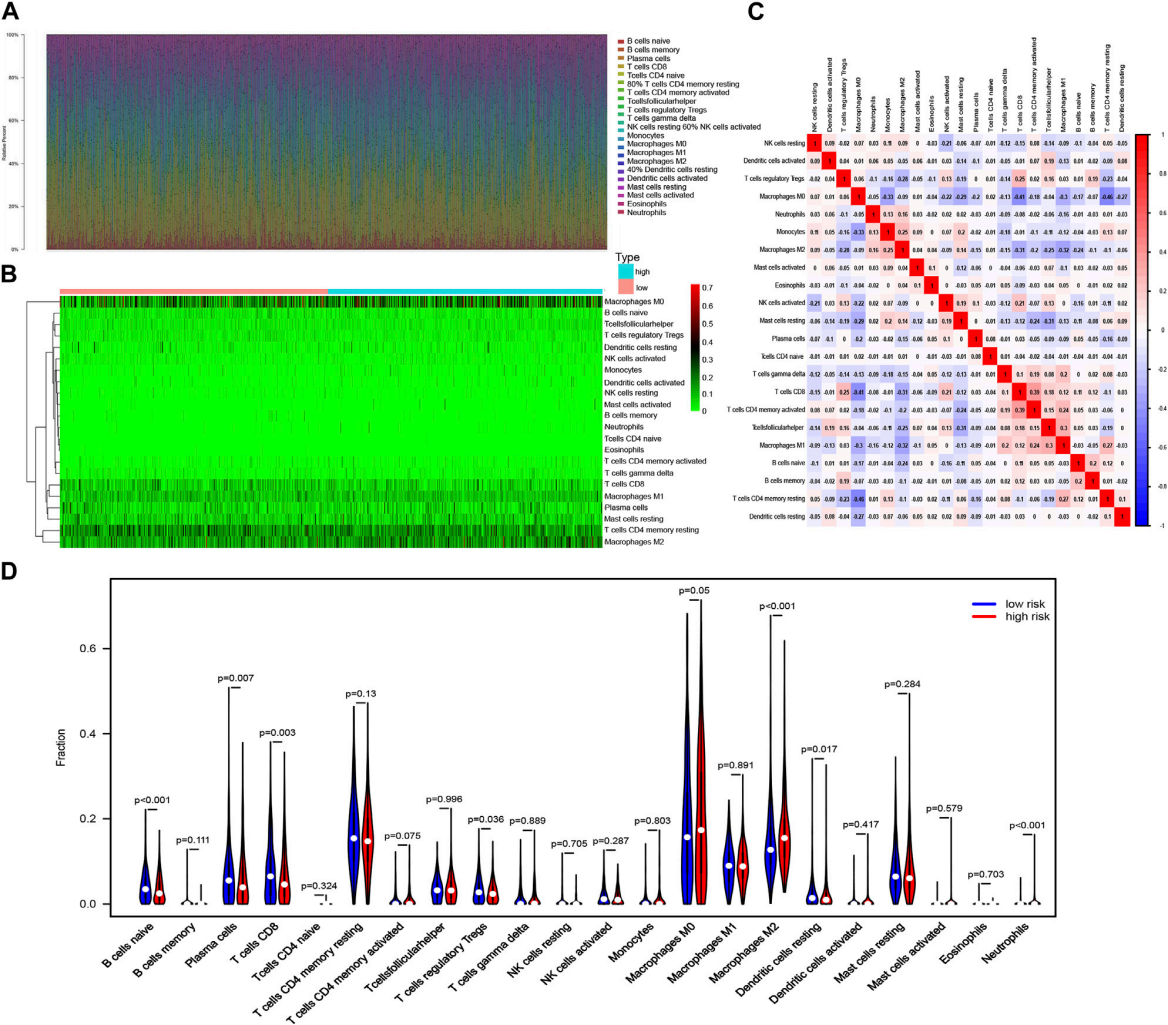
Immune cell infiltration landscape in breast cancer. **(A)** Bar plot of the tumor-infiltrating cell proportions. **(B)** Heatmap of the tumor-infiltrating cell proportions. **(C)** Correlation matrix of immune cell proportions. **(D)** Violin plot of the different proportions of tumor-infiltrating cells between the high-risk group and the low-risk group.

The proportions and correlation matrix of immune cell proportions in the training cohort are presented in Figures 6A–C. A violin plot was drawn to show the different proportions of the 22 types of tumor-infiltrating cells between the high- and low-risk groups in the training cohort. Anti-tumor immune cells such as CD8^+^ T cells and regulatory T cells (Tregs) were downregulated in the high-risk group, whereas cancer-promoting cells such as M2 macrophages were upregulated in the high-risk group (Figure 6D), which indicated that patients with BC in the high-risk group had anti-cancer immune deficiency. More importantly, immune cell infiltration was proven to reflect the efficacy of immunotherapy. CD8^+^ T lymphocytes are effector cells of immune checkpoint blockage therapy (ICB), and the exhaustion of CD8^+^ T cells leads to inefficiency of ICB in patients with breast cancer (Rahim et al., 2023). Tregs suppress anti-cancer immunity, thereby hindering protective immunosurveillance of tumors. Targeting tumor-infiltrated Tregs, such as depletion of Tregs, and targeting immune checkpoint on Tregs or skewing Tregs toward anti-tumor immunity phenotype were important directions for breast cancer immunotherapy (Li et al., 2020).

Among patients with HER2− BC, anti-tumor immune cells, such as CD8^+^ T cells and resting dendritic cells, were obviously downregulated in the high-risk group, while cancer-promoting cells, such as CD4^+^ T memory resting cells, were upregulated in the low-risk group (Supplementary Figure S6). Anti-tumor Tregs were also downregulated in the high-risk group of patients with HER2+ BC (Supplementary Figure S6). Resting anti-tumor immune dendritic cells were also downregulated in the high-risk group among patients with HR+ BC, while cancer-promoting cells, such as M2 macrophages and neutrophils, were upregulated in the low-risk group (Supplementary Figure S6). B and T lymphocyte attenuator (BTLA) is one of the most important co-signaling molecules (Ning et al., 2021).

### 3.7 Cuproptosis-related comprehensive model in predicting the sensitivity of conventional treatment in the training cohort

**FIGURE 7:**
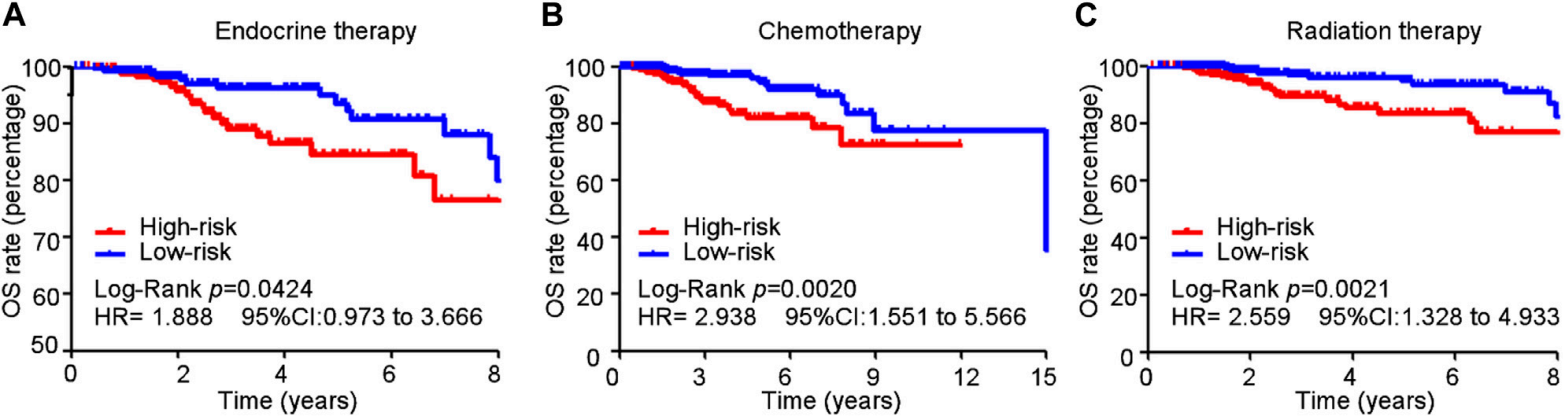
Cuproptosis-related comprehensive model in predicting the sensitivity of conventional treatment in the training cohort. **(A–C)** BC patients received different therapy in the high-risk group suffered a poorer survival than those in the low-risk group (endocrine therapy: HR = 1.888, 95% CI 0.973–3.666, *p* = 0.042; chemotherapy: HR = 2.938, 95% CI 1.551–5.566, *p* = 0.0002; radiation therapy: HR = 2.559, 95% CI 1.328–4.933, *p* = 0.0021).

In the training cohort, 513, 575, and 518 of 1,054 patients with BC received endocrine, chemotherapy, and radiation therapy, respectively. Patients with BC who received different therapies in the high-risk group exhibited poorer survival than those in the low-risk group (endocrine therapy: HR = 1.888, 95% CI 0.973–3.666, *p* = 0.042; chemotherapy: HR = 2.938, 95% CI 1.551–5.566, *p* = 0.0002; radiation therapy: HR = 2.559, 95% CI 1.328–4.933, *p* = 0.0021) (Figures 7A–C).

### 3.8 Cuproptosis-related comprehensive model in predicting the sensitivity of CDK4/6 inhibitor and PARP inhibitor

**FIGURE 8:**
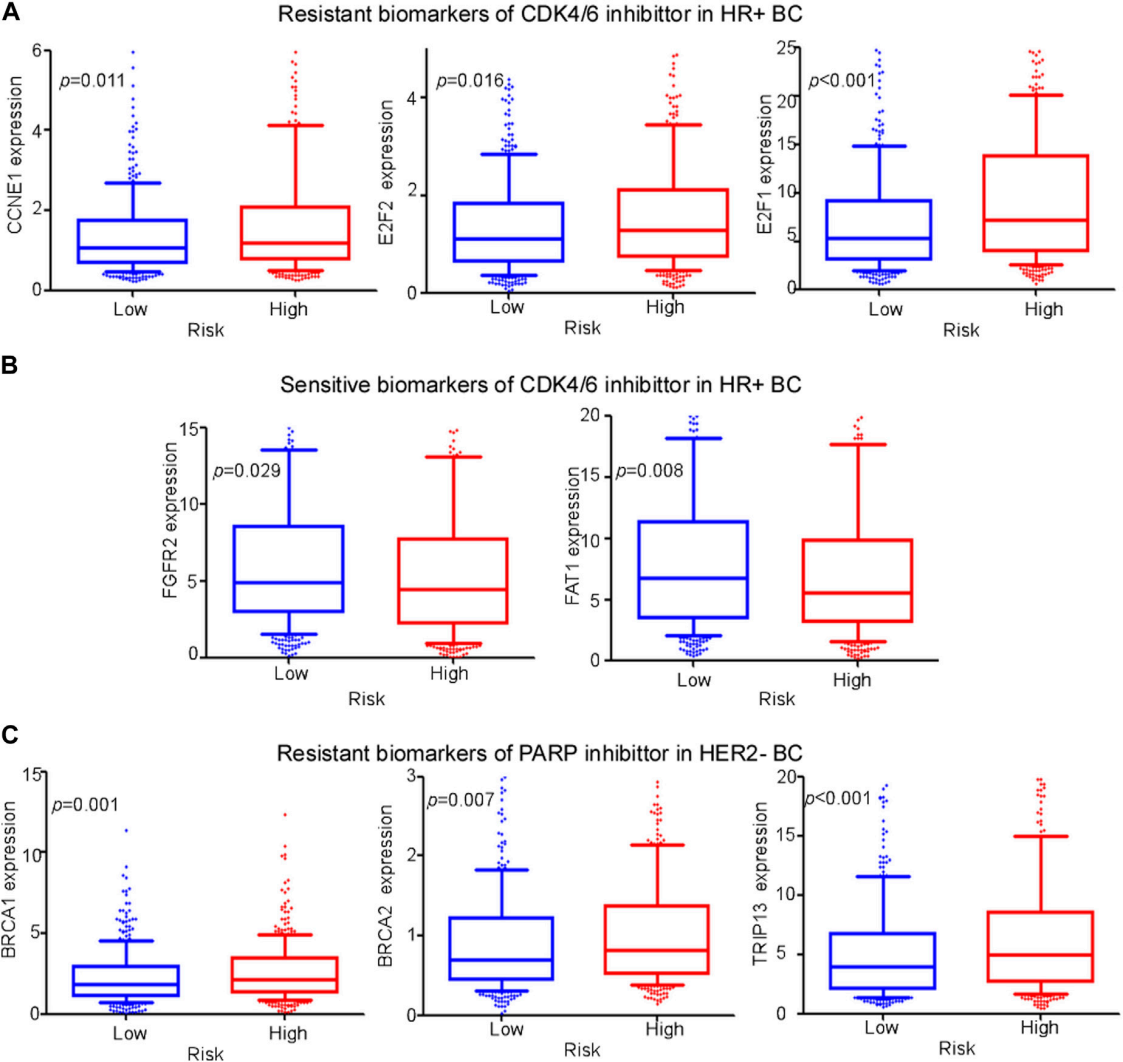
Evaluation of emerging treatments. **(A,-B)** Expression levels of different biomarkers of resistance and sensitivity of CDK4/6 inhibitors between high- and low-risk groups among HR+ BC patients. **(C)** Expression levels of different biomarkers of resistance of PARP inhibitors between high- and low-risk groups among HER2− BC patients.

Pharmacological inhibitors of cyclin-dependent kinases 4 and 6 (CDK4/6) and PARP, two emerging treatments, are effective in patients with HR+ and HER2− BC, respectively. In this study, we developed a comprehensive cuproptosis-related model to evaluate the therapeutic effects of these treatments. In previous research, a high expression of CCNE1, E2F1, and E2F2 and lower expression of FAT1 and FGFR2 led to poorer sensitivity to CDK4/6 inhibitors, while lower expression of BRCA1, BRCA2, and TRIP13 contributed to higher sensitivity to the PARP inhibitor (Rose et al., 2020; Bui et al., 2022). In our study, compared with low-risk patients, high-risk patients exhibited a higher expression of resistant biomarkers (CCNE1, E2F1, and E2F2) (Figure 8A) and lower expression of sensitive biomarkers of CDK4/6 inhibitors (FAT1 and FGFR2) (Figure 8B). In addition, a higher expression of resistance biomarkers (BRCA1, BRCA2, and TRIP13) was detected in high-risk patients than in low-risk patients (Figure 8C). The expression of these genes indicated that patients with BC in the low-risk group were more suitable for PARP inhibitor treatment, and patients in the high-risk group had a higher risk of drug resistance.

Pharmacodynamics experiments were performed to test whether the cuproptosis-related lncRNAs were associated with drug efficacy of CDK4/6 inhibitors or PARP inhibitor. As presented in Supplementary Figure S8, silencing YTHD-AS1 in MCF7 cells could reduce IC_50_ of CDK4/6 inhibitor, while silencing TOLLIP-AS1 increased IC_50_. In addition, silencing YTHD-AS1 or NIFK-AS1 in MDA-MB-231 cells could reduce IC_50_ of PARP inhibitors. The aforementioned data suggested that the cuproptosis-associated-lncRNA model potentially predicted the sensitivity of CDK4/6 inhibitor or PARP inhibitors.

## 4 Discussion

Female BC is the leading cause of global cancer incidence and the fifth leading cause of cancer-related mortality worldwide, as of 2020 (Sung et al., 2021). As an essential trace element, excess copper is toxic, and it leads to cell death by the TCA cycle, which is associated with tumor progression. Few cuproptosis-related lncRNA signatures have been constructed for predicting the survival and immune microenvironment of patients with BC (18, 19); however, they did not validate their model in an external dataset or predict the therapeutic efficacy of emerging therapies in patients with BC. In our study, we combined cuproptosis-related lncRNAs and clinical characteristics to predict the prognosis of BC. These markers exhibited a high predictive accuracy, and they can serve as a potential indicator of resistance to PARP inhibitors and CKD4/6 inhibitors.

Among the six cuproptosis-related lncRNAs, YTHDF3.AS1 has not been previously reported, whereas the other five lncRNAs have been reported to affect tumor prognosis. Overexpression of the lncRNA NIFK-AS1 inhibited the proliferation, migration, and invasion of endometrial cancer cells by suppressing the M2-like polarization of macrophages (Zhou et al., 2018). LncRNA TP53TG1 suppresses the growth and metastasis of tumor cells by regulating the PRDX4/beta-catenin pathway (Chen et al., 2021b). The lncRNA TOLLIP.AS1 was found to be a protective factor against BC in a previous lncRNA model (Shi et al., 2022b). LINC00839 promotes the proliferation, migration, invasion, and apoptosis of neuroblastoma cells, indicating lower overall survival (Yang et al., 2021). The lncRNA OTUD6B-AS1 was also found to promote therapeutic resistance in TNBC, resulting in poor prognosis (Li et al., 2021). The lncRNAs YTHDF3-AS1, LINC00839, and OTUD6B-AS1 played an important role in promoting proliferation and metastasis of BC cells, according to our biofunctional experiments. Therefore, the six cuproptosis-related lncRNA signatures identified by us are partly convincing.

The role of cuproptosis in cancer is complex; nevertheless, it has been receiving increasing attention. Patients with BC have been reported to have excess copper, a trace element, which induces cell death and exhibits the potential to promote tumor progression (Blockhuys and Wittung-Stafshede, 2017; Blockhuys et al., 2020). Copper-related therapeutic agents, such as disulfiram, significantly inhibited tumor growth without significant toxicity, but caused apoptosis only in tumor cells by inhibiting the levels of reactive oxygen species among inflammatory BC cells and contributing to therapy sensitivity (Allensworth et al., 2015). Cuproptosis-related lncRNAs in BC were screened and combined with clinical data to develop a comprehensive cuproptosis-related model to predict BC prognosis. Our model showed a high predictive value in the training cohort and the other two validation cohorts, and a strong agreement was detected between the actual and predicted OS rates of our comprehensive model. In contrast, our model provided corresponding points for each predictive factor, offering a precise and simple calculation method for predicting the 1-, 3-, and 5-year survival probabilities of OS.

In our study, the CIBERSORT algorithm was used for exploring the relationship between apoptosis and immune cell infiltration in breast cells. Compared with the low-risk group, the high-risk group showed a significantly reduced number of anti-tumor-infiltrating immune cells, such as CD8^+^ T cells and Tregs but an increased number of M2 macrophages, which are immune cells promoting tumor proliferation and metastasis (Togashi et al., 2019; Xia et al., 2020; Reina-Campos et al., 2021). Thus, cuproptosis is associated with the proportion of tumor-infiltrating immune cells in BC. Moreover, compared with the low-risk group, the high-risk group showed a lower expression of the immune checkpoint molecule BTLA among patients with HER2+, HER2−, and TNBC and a lower expression of B7H3 among patients with HER2+ BC, indicating that patients with BC in the high-risk group may tend to be immunologically “cold” and may gain little benefit from immunotherapy.

CDK4/6 and PARP inhibitors are two emerging treatments for BC. However, BC is heterogeneous, and not all patients are sensitive to these inhibitors. CDK4/6 inhibitors have become the first highly specific CDK inhibitors approved for cancer treatment. Endocrine therapy in combination with CDK4/6 inhibitors has been proven to prolong survival of patients with metastatic HR+ and HER2− BC (Chong et al., 2020). CCNE1 amplification leads to acquisition of resistance to CDK4/6 inhibition, owing to the bypass of cyclin D1-CDK4/6 dependency (Herrera-Abreu et al., 2016), which is highly expressed in the high-risk group among patients with HR+ or HER2− BC. As a tumor suppressor, the loss of FAT1 promotes resistance to CDK4/6 inhibitors via the Hippo pathway (Li et al., 2018), which was expressed at low levels in the high-risk group among patients with HER2− BC. Thus, patients with the HR+ or HER2− phenotype in the high-risk group were more likely to be resistant to CDK4/6 inhibitors, and we inferred that our cuproptosis-related model facilitates the identification of patients with BC who are more susceptible to CKD4/6 inhibitor resistance. A previous study showed that gene mutations of *BRCA1* and *BRCA2* are related to a high risk of BC occurrence (Antoniou et al., 2003); however, BRCA1- and BRCA2-deficient tumors are sensitive to PARP1 inhibitors, and patients with these types of tumors gain more benefit from this treatment (D’Andrea, 2018). Lower expression of BRCA1 or BRCA2 was observed in the low-risk group, indicating that low-risk patients with BC were more suitable for PARP inhibitor treatment. TRIP13 overexpression is common in BRCA1-deficient cancers, which leads to PARP-inhibitor resistance through conformational changes in REV7 and reduces drug sensitivity (Clairmont et al., 2020). Additionally, TRIP13 overexpression was observed in the high-risk group. MUS81 nuclease inhibition restores DNA-replication-fork protection but not repair, leading to PARP inhibitor resistance in patients with mutant BRCA2 (Schlacher, 2017). Low MUS81 expression was observed in the high-risk group. Thus, patients expressing the HR+ or HER2− phenotype in the high-risk group were more likely to be resistant to PARP inhibitors, and we inferred that our cuproptosis-related model can aid in identifying patients who are more liable to PARP-inhibitor resistance.

Our study has some limitations. First, the expression of cuproptosis-related lncRNAs in this study was based on TCGA and GEO public databases, lacking verification of prospective, multicenter, and real-world data by qRT-PCR or deep sequencing. Second, our study only revealed a relationship between cuproptosis-related lncRNAs and OS in patients with BC. The underlying molecular mechanisms are not fully understood and require further investigation. Third, the potential of the cuproptosis-related comprehensive model for evaluating tumor immunity was only tested using the CIBERSORT algorithm, and further experiments are needed in the future. Finally, we evaluated the efficacy of the cuproptosis-related comprehensive model by analyzing the expression of related biomarkers. However, further investigation is required.

In summary, we successfully constructed a comprehensive cuproptosis-related model that demonstrated that cuproptosis-related lncRNAs are associated with breast cancer prognosis. In addition, our model showed that combining clinical characteristics with cuproptosis-related lncRNAs markedly increased the predictive value of prognosis in patients with BC. Moreover, our model exhibited the potential to indicate resistance to PARP and CKD4/6 inhibitors.

## Data Availability

The datasets presented in this study can be found in online repositories. The names of the repository/repositories and accession number(s) can be found in the article/Supplementary Material.

## Abbreviations

BC, breast cancer; BTLA, B and T lymphocyte attenuator; CSGs, cancer susceptibility genes; GSEA, gene set enrichment analysis; HER2+ BC, HER2-positive breast cancer; HER2− BC, HER2-negative breast cancer; HR+ BC, hormone receptor-positive breast cancer; OS, overall survival; PARP, poly (ADP-ribose) polymerase; TCA, tricarboxylic acid; TNBC, triple-negative breast cancer.
