# Supplementary material for: Cuproptosis-related lncRNAs potentially predict prognosis and therapy sensitivity of breast cancer
Source: Front Pharmacol. 2023 Jul 17;14:1199883. doi: 10.3389/fphar.2023.1199883 (PMC10390311; doi:10.3389/fphar.2023.1199883)
Supplement: Supplementary file 1 [file DataSheet1.DOCX]

**Supplementary figures**

**
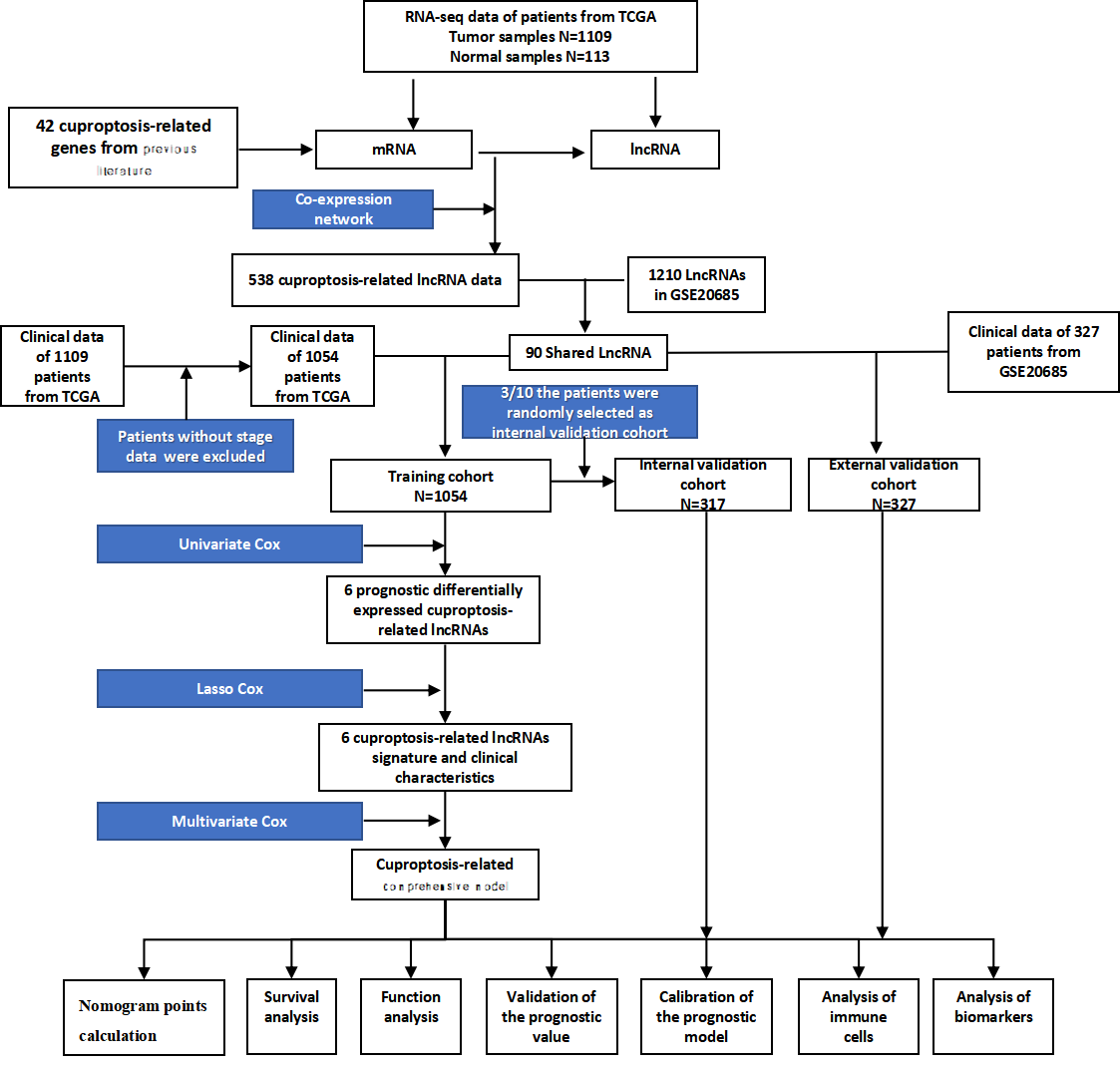
**

**Figure S1. The flow chart of our study.**


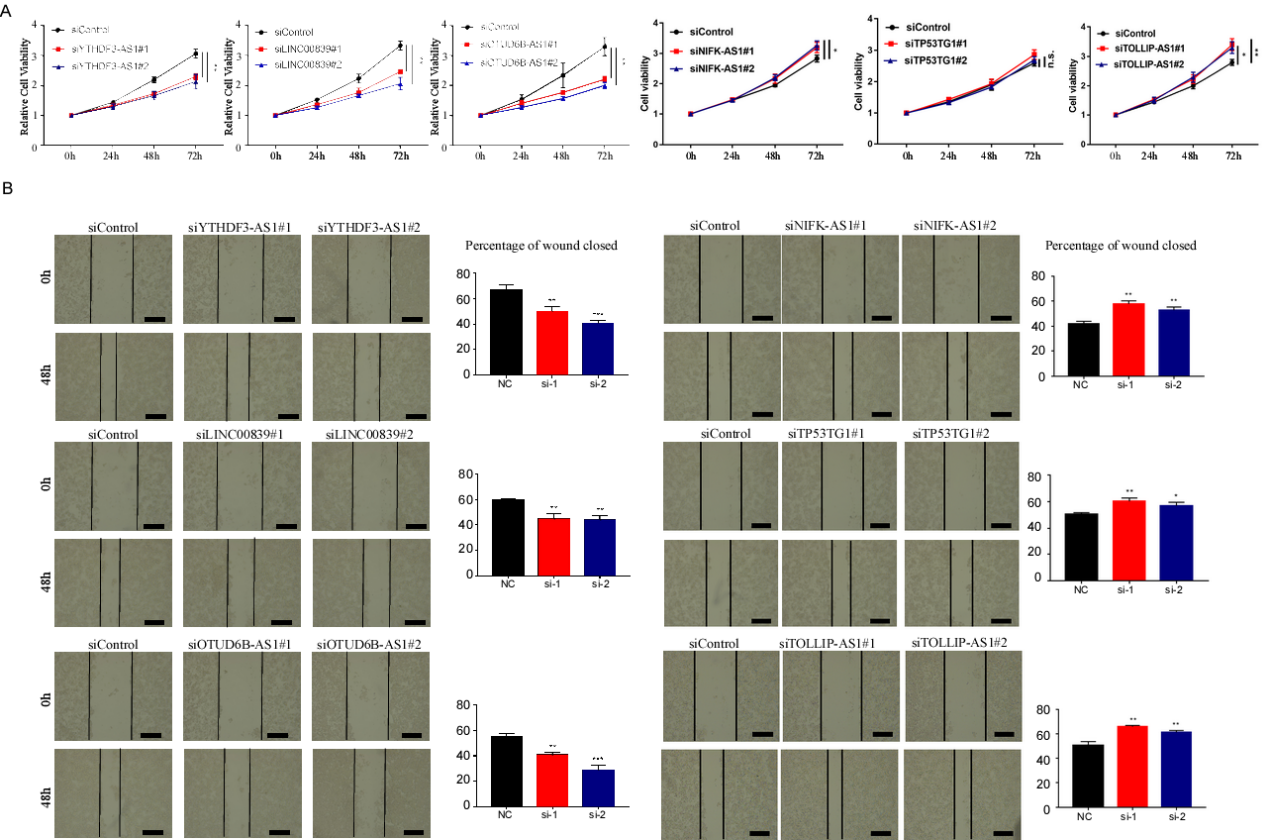


**Figure S2. Bio-functional of selected lncRNA in breast cancer cells**：

1. CCK-8 kit assay indicated that the proportion of living cells were decreased after silencing selected lncRNAs in MCF-7 cells, scale bar - 100μm.
2. Wound healing assay indicated that the migration vitality was decreased after silencing silencing selected lncRNAs in MCF7.

**P* < 0.05, ***P* < 0.01, ****P* < 0.001, n.s. no significance, according to Student’s t test.


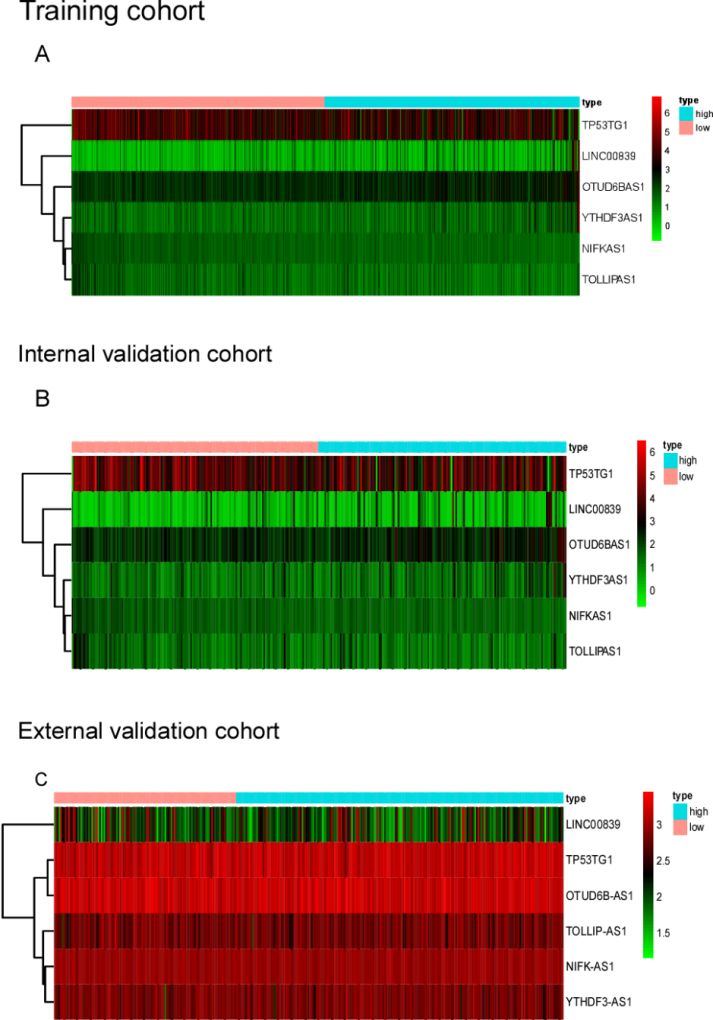


**Figure S3. Gene expression heat map in the training cohort and validation cohort:**

1. Gene expression heat map in the training cohort.
2. Gene expression heat map in the internal validation cohort.
3. Gene expression heat map in the external validation cohort.


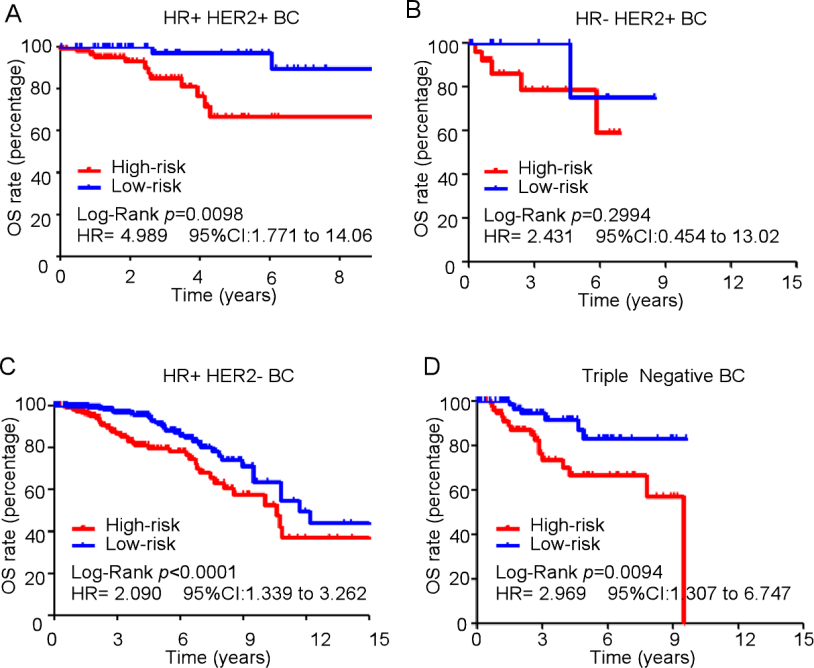


**Figure S4. Overall survival of BC patients with high- and low risk:**

Kaplan-Meier curves for OS in all patients with HR+HER2- BC (A), HR-HER2+ (B), HR2+HER2+ BC (C) and TNBC(D) subtype.

**
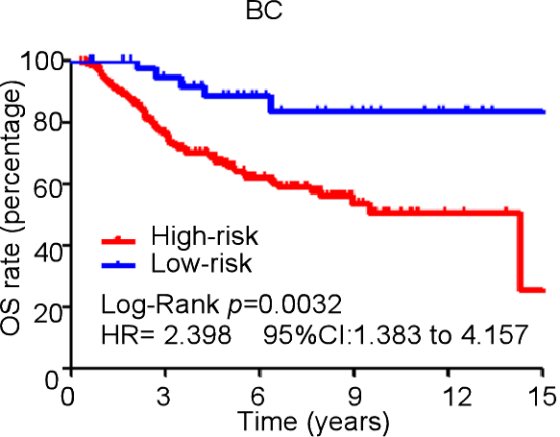
**

**Figure S5. Overall survival of BC patients with high- and low risk in GSE21653 dataset.**


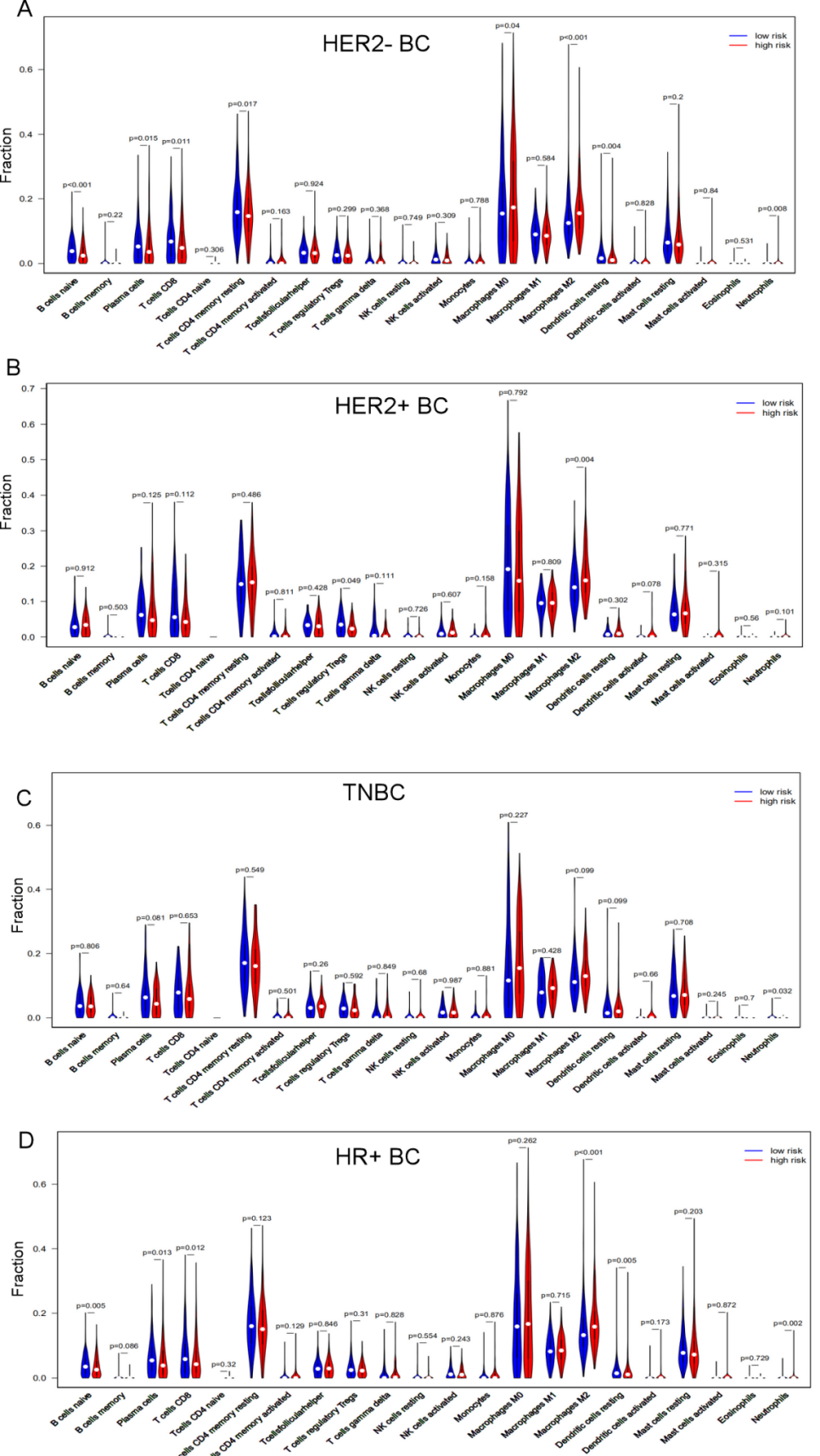


**Figure S6. The immune cell infiltration landscape in breast cancer**

1. Violin plot of the different proportions of tumor-infiltrating cells between high-risk group and low-risk group in HER2- subgroup.
2. Violin plot of the different proportions of tumor-infiltrating cells between high-risk group and low-risk group in HER2+ subgroup.
3. Violin plot of the different proportions of tumor-infiltrating cells between high-risk group and low-risk group in TNBC subgroup.
4. Violin plot of the different proportions of tumor-infiltrating cells between high-risk group and low-risk group in HR+ subgroup.

**
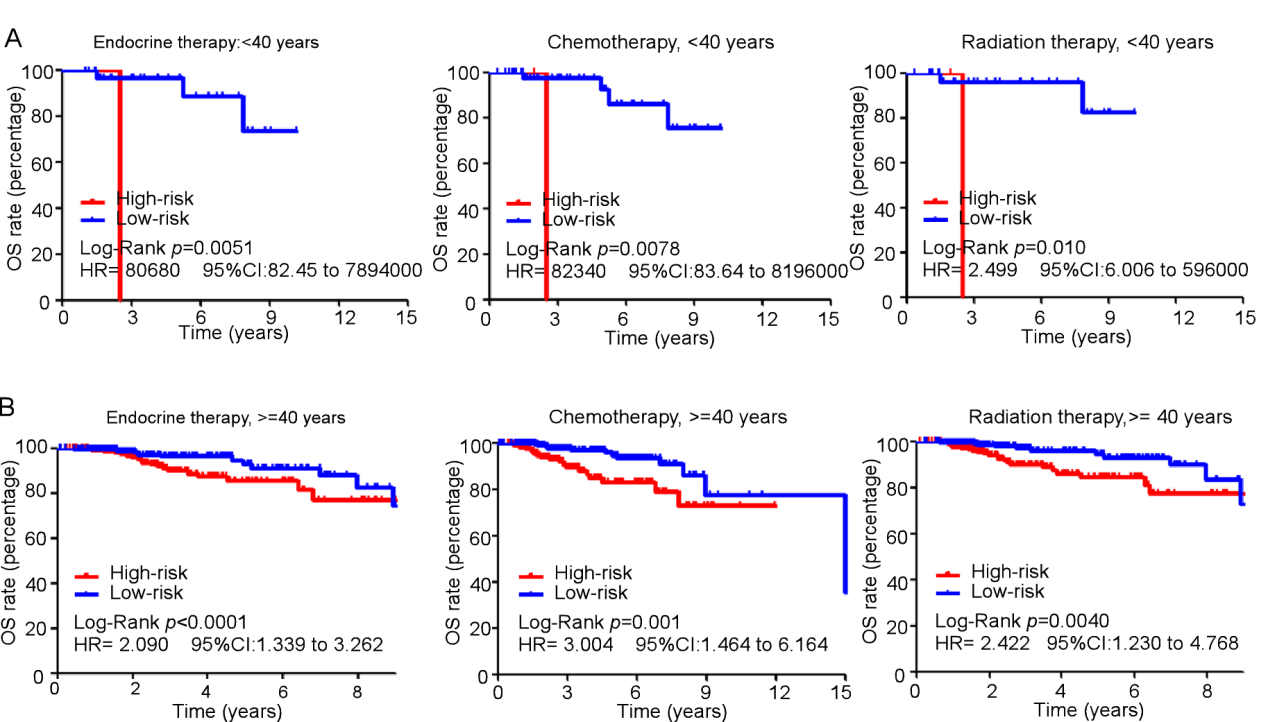
Figure S7. Cuproptosis-related comprehensive model in predicting sensitivity of conventional treatment in training cohort.**

1. OS in high- and low-risk patients (Aged <40 years) under endocrine therapy(left), chemotherapy (middle) and radiation therapy(right).
2. OS in high- and low-risk patients (Aged >=40 years) under endocrine therapy(left), chemotherapy (middle) and radiation therapy(right).


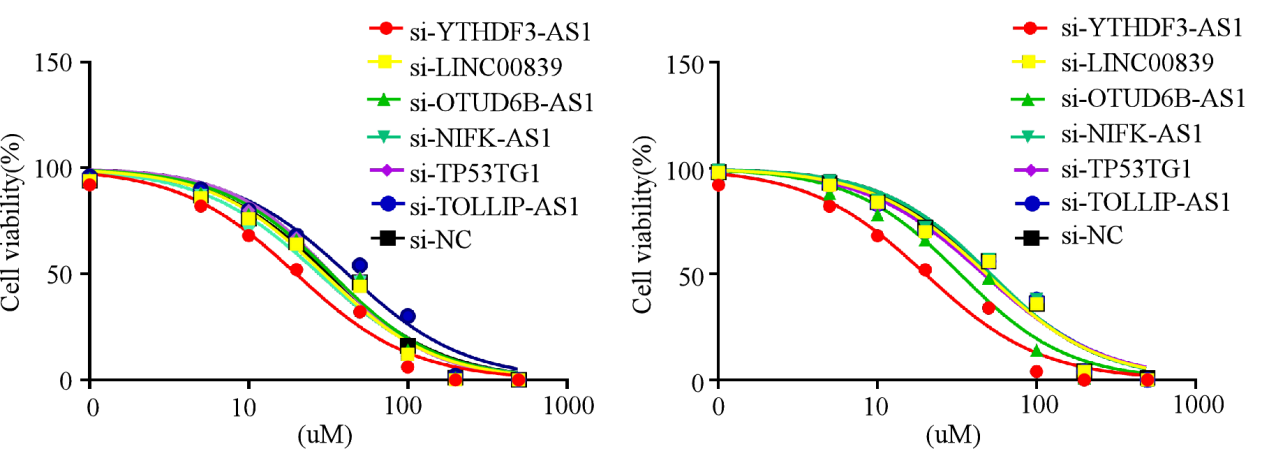


**Figure S8. The regulatory role of selected lncRNAs in CDK4/6 inhibitor and PARP inhibitor.**

1. IC50 of CDK4/6 inhibitor (Palbociclib) in MCF7 cells after silencing selected lncRNAs. (IC50: si-NC 31.71 uM; si-YTHDF3-AS1 17.59 uM; si-LINC00839 29.71 uM; si-OTUD6B-AS1 33.19 uM; si-NIFK-AS1 27.86 uM; si-TP53TG1 33.31 uM; si-TOLLIP-AS1 49.65 uM.)
2. IC50 of PARP inhibitor (Olaparib) in MDA-MB-231 cells after silencing selected lncRNAs. (IC50: si-NC 49.9 uM; si-YTHDF3-AS1 20.07 uM; si-LINC00839 48.6 uM; si-OTUD6B-AS1 33.19 uM; si-NIFK-AS1 51.66 uM; si-TP53TG1 46.5 uM; si-TOLLIP-AS1 49.11 uM.)
